# Real time monitoring of oxygen uptake of hepatocytes in a microreactor using optical microsensors

**DOI:** 10.1038/s41598-020-70785-6

**Published:** 2020-08-13

**Authors:** Christian Gehre, Marie Flechner, Sarah Kammerer, Jan-Heiner Küpper, Charles Dominic Coleman, Gerhard Paul Püschel, Katja Uhlig, Claus Duschl

**Affiliations:** 1 Branch Bioanalytics and Bioprocesses (IZI-BB), Fraunhofer-Institute for Cell Therapy and Immunology, Potsdam, Germany; 2 Faculty of Science, Brandenburg University of Technology Cottbus-Senftenberg, Senftenberg, Germany; 3 Department of Nutritional Biochemistry, Institute of Nutritional Science, University of Potsdam, Nuthetal, Germany

**Keywords:** Biophysics, Biotechnology, Cell biology, Drug discovery

## Abstract

Most in vitro test systems for the assessment of toxicity are based on endpoint measurements and cannot contribute much to the establishment of mechanistic models, which are crucially important for further progress in this field. Hence, in recent years, much effort has been put into the development of methods that generate kinetic data. Real time measurements of the metabolic activity of cells based on the use of oxygen sensitive microsensor beads have been shown to provide access to the mode of action of compounds in hepatocytes. However, for fully exploiting this approach a detailed knowledge of the microenvironment of the cells is required. In this work, we investigate the cellular behaviour of three types of hepatocytes, HepG2 cells, HepG2-3A4 cells and primary mouse hepatocytes, towards their exposure to acetaminophen when the availability of oxygen for the cell is systematically varied. We show that the relative emergence of two modes of action, one NAPQI dependent and the other one transient and NAPQI independent, scale with expression level of CYP3A4. The transient cellular response associated to mitochondrial respiration is used to characterise the influence of the initial oxygen concentration in the wells before exposure to acetaminophen on the cell behaviour. A simple model is presented to describe the behaviour of the cells in this scenario. It demonstrates the level of control over the role of oxygen supply in these experiments. This is crucial for establishing this approach into a reliable and powerful method for the assessment of toxicity.

## Introduction

Presently, there is a great demand for in vitro test systems for the elucidation of the mode of action of drug candidates and in particular for the assessment of toxic effects of compounds^1–3^. The reasoning for this demand is diverse but several points are crucial in this context: Tests based on animals do not fully reflect all the aspect of human nature, animal tests are expensive and the throughput achievable is rather small^4,5^. Finally, ethical concerns have a growing impact on the overall discussion of this subject. This is widely acknowledged and as a result, enormous efforts have been made in recent years to develop methods that do not only give meaningful results but are robust in order to enable a thorough validation. The bulk of recent work has been dedicated to two key components, the cell models^6–9^ and the formats of the reactors^10–12^, the cells are implemented in. The former is motivated through the necessity to use cells that mimic essential functional details of the related tissue or organs. The format of the cell reactor must enable three major functions: Firstly, it has to provide an environment that is able to maintain status and the vitality of the cells. Secondly, the fluidic system should ensure the supply of medium, the availability of important soluble molecular factors and the controlled immersion of cells with the compounds of interest. Thirdly, access of powerful characterization methods to the cells is crucial for a detailed analysis of their behaviour. In this context, two approaches must be distinguished: Invasive characterization methods are much in use. Although they may provide detailed information about the response of cells, this information describes only a singular point in time and hence it is difficult and/or tedious to elucidate processes that evolve in time. A complementary approach is based on continuous noninvasive monitoring of parameters that are closely related to cell behaviour. This approach produces an abundance of data. Some cases have already been reported in which it helped to decipher cellular mechanism hardly accessible to conventional endpoint measurements, however its value for replacing well established and validated in vitro test formats has still to be demonstrated^13,14^.

A very prominent part of work in the field is dedicated to the development of so-called organ-on-a-chip devices^15^. By making use of the advancements of microtechnology in recent years, the focus of this approach lies in optimising the microenvironment of the cells on the chip in order to mimic important features of the human physiology. This comprises the fluidic supply on one hand and on the other hand the establishment of microstructures that can guide the growth of specific tissue-like cell assemblies^16–18^. Considerable progress has already been achieved in this respect. Sophisticated fluidic infrastructures do not only supply the cells with precise amounts of medium, signal molecules and compounds to be tested, they also connect cells of different origin and thereby allow the emulation of the exchange of signals between different organs in the human body^19^.

Despite all these advances, there remain considerable challenges when it comes to the analysis of cell response and development in real time in order to gather quantitative information on these processes^20^. This problem has been mainly addressed using electrochemical methods so far, for example, for monitoring pH or changes of glucose concentration in the medium^21,22^. However, these methods are very difficult to implement and are particularly prone to drift of signals and to interference with the many charged entities present in complex biological samples. However, recently it has been demonstrated that long term monitoring of changes of oxygen and lactate concentration caused by the metabolism of single spheroids can be reliably measured^23^.

Optical sensors for the measurement of oxygen concentrations provide a valuable alternative to electrochemical methods and offer a number of unique features when it comes to the monitoring of cellular processes^24,25^. The advances in employing optical methods for measuring oxygen and related parameters has been substantial over recent years (see, for example the comprehensive reviews of Wang and Wolfbeis and of Quaranta et al.)^26,27^. Here we present results that are obtained from real time monitoring of the oxygen consumption of hepatocytes of three different origins using a robust optical detection scheme. The oxygen consumption of the cells is a measure of their metabolic activity and provides crucial information on the vitality of the cells^28^. The method employed here exploits the oxygen sensitivity of the emission of Ruthenium-Phenanthroline^29^. The chromophore is immobilised into polymer beads, which provide a very stable matrix. The phosphorescence lifetime of the chromophore is directly related to the local oxygen concentration and can easily be measured using intensity modulated excitation light and a phase sensitive detection scheme. As the signal does not depend on the phosphorescence intensity, the requirements in respect to optical alignment and precision are rather relaxed enabling a very robust measurement scheme. In our reactor, cells and sensor beads are placed into small wells where they are immobilised using a collagen matrix. By carefully controlling the oxygen supply through the medium, the number of cells that consume oxygen and the locations of sensor beads in respect to those of the cells, the dynamic range of this measurement regime can be fully exploited for monitoring the metabolic activity of cells. This approach also enables us to adjust oxygen concentrations that correspond to the physiological conditions in the liver. We do not aim at an utmost miniaturisation with this system, simply because a sufficient number of cells are needed to consume sufficient oxygen and hence we consider our system to be a reactor rather than an organ-on-a-chip. It has been shown that this approach can provide important information on the cellular response of the hepatocyte cell line HepG2 to the exposure to various drugs or compounds known to be hepatotoxic. In the case of the broadly used analgesic and antipyretic acetaminophen, the measurement of oxygen uptake as a function of time revealed two independent time constants indicating for the first time that besides the well-described CYP-dependent toxin mechanism of action a direct mechanism of action on the mitochondrial respiration is present^13^. The highly controlled perfusion of the cells in this reactor allows to maintain cell viability to more than 4 weeks^13,14^.

In this work, we explore and identify conditions in which optimal use of oxygen microsensor beads for the monitoring of oxygen consumption of small cell samples can be made. In particular, in this contribution we focus on the investigation of how cells respond to their exposure to various concentration of acetaminophen when different levels of oxygen supply are present. For this, we employed hepatocytes, the most important cell type for the assessment of toxicity of compounds, from three different sources: The cell line HepG2 is a widely used test system but shows a rather small capability to biotransform drugs in respect to primary hepatocytes, which limits its relevance for toxicity tests. The cell clone HepG2-3A4 overexpresses the enzyme CYP3A4 in comparison to HepG2 parental cells^30^. CYP3A4 is considered to be the most important enzyme in drug metabolism. It metabolises approximately 50% of all clinically used drugs. However its role in metabolising acetaminophen seems to be less prominent in comparison CYP2E1 and CYP1A2^31^. Finally, primary cells from mice are also included as we suppose that their CYP expression exceeds the ones of the cell lines used. Hence, we can make use of three cell types that all show different levels of metabolization of acetaminophen. In addition, the primary cells are known to have a considerably higher oxygen consumption rate, providing us with an additional parameter to vary for characterising our system. In an effort to ensure a high level of control over the microenvironment of the cells, an improved version of the microbioreactor introduced recently has been employed.

## Materials and methods

### Microreactor design and manufacturing

Each microreactor consists of a cell carrier with three channels, which are open towards the top and are 20 mm long, 0.6 mm high and 2 mm wide. These allow the examination of three different measuring conditions on one unit. Three microcavities per channel are embedded in the channel bottom with a diameter of 1.5 mm and a height of 0.3 mm. The cell carriers were fabricated by soft lithography using the elastomer PDMS (SYLGARD 184, Dow Corning, USA), which were moulded in casting stations and cured for two hours at 90 °C. The bottom of the cell carrier was sealed with a cover glass to prevent gas exchange from the environment to the liquid in the microchannel. To bond the cell carrier to the PMMA channel plate, double-side adhesive tape (PSA, 3 M Medica, Germany) was used. The PMMA channel plate was fabricated by milling and connects the microchannels to the tubing (FEP-Tubing, Techlab, Germany) via ¼” threads and fluidic adapters (Flangeless Male Nut, Techlab, Germany).

### Cell culture

HepG2 hepatocytes (ACC 180, DSMZ, Germany) were cultivated in DMEM containing HEPES (25 mM), FCS (10%), penicillin/streptomycin (1%) and l-glutamine (2 mM, all Biochrom, Germany).

For cultivation of HepG2-3A4 (source BTU Cottbus-Senftenberg, Germany)^30^ the medium of HepG2 was modified by supplementing 3 µg/ml blasticidin (AppliChem, Germany). Both cell lines were cultured at 37 °C and 5% CO_2_. For subculture, the cells were rinsed twice with PBS and incubated with 0.25% Trypsin/EDTA (Biochrom, Germany) for 7 min at 37 °C.

Primary mouse hepatocytes were isolated from C57/BL6 mice as described previously^32^. Briefly, the liver was first perfused with calcium- and magnesium-free Hank's balanced salt solution containing 1 mM EDTA followed by a perfusion with Hank's balanced salt solution containing 0.016 mg/ml Liberase (Roche, Germany). Hepatocytes were separated from non-parenchymal cells and debris by density centrifugation with 58% Percoll in phosphate buffered saline.

The principles of laboratory animal care were followed. Treatment of the animals followed the German animal protection laws and was performed with permission of the state animal welfare committee (LUGV Brandenburg, 2347-A-16-2-2017).

### Cell seeding procedure

4 × 10^6^ freshly trypsinized or isolated cells were centrifuged for 5 min at 300×*g*. Afterwards, the supernatant was removed and the cells were resuspended in 15 μl of ice-cold solution of collagen type I (BD Biosciences, USA) and 100 µg CPOx-Beads (Colibri Photonics, Germany). 0.8 µl of the cell/bead suspension were pipetted in each cavity of the ice cooled cell carrier to achieve a cell concentration of 1 × 10^5^ cells per cavity for HepG2 and HepG2-3A4. The primary mouse hepatocytes were seeded into cavities directly after isolation. In contrast to the cell lines, the cell number of primary mouse hepatocytes had to be adjusted to 3 × 10^4^ cells per cavity due to their high oxygen consumption rates. To alter the amount of cells per cavity, we have regulated the cell concentration with different amounts of collagen. The cell carrier was then incubated for 8 min at 37 °C to polymerize the collagen. Afterwards the cell carrier was ready for connection to the PMMA channel plate. Oxygen measurements were started approximately two hours after seeding.

### Real-time oxygen measurements

For real-time oxygen detection, an OPAL (Colibri Photonics, Germany) was connected to a fully automated Olympus microscope (IX81, Olympus, Japan). The 532 nm LED of the OPAL and a filter cube with 531/40 nm (excitation), 607/70 nm (emission) were inserted in the optical light path during measurements. This setup allows quantifying the oxygen concentration via lifetime-based luminescence quenching of a ruthenium-phenanthroline-based phosphorescence dye loaded in microbeads (CPOx-50-RuP). The oxygen measurements were performed by averaging ten seconds exposures of sensor beads in each cavity using a 20 × objective. The cavities were measured consecutively using an automatic routine. All time course experiments were repeated at least three times. The measurement data was further processed and graphically displayed using OriginPro 2015G, 64-bit (https://www.originlab.com).

### Microfluidic setup

The microreactors were perfused using syringe pumps (sp230iw, WPI, USA). The syringes were connected at the outlets of the reactor. Two medium reservoirs were connected to each reactor channel via electrically controllable valves (3/2-way flipper valve, Buerkert, Germany), one reservoir contained medium and the other medium with acetaminophen (APAP, Sigma-Aldrich, Germany). The syringes pulled the medium out of the target reservoirs at a flow rate of 20 µl/min in order to supply the cells continuously with oxygen-rich medium. To study the effect of APAP on hepatocytes, the cells were exposed to 0, 1, 4, 8, 12.5, 16 mM for 12, 24 or 48 h. Before and after APAP exposure the cells were continuously supplied with medium. To ensure optimal cell culture conditions, the microreactor and the medium reservoirs are placed into a microscope climate chamber (Evotec, Germany).

## Results

**Figure 1:**
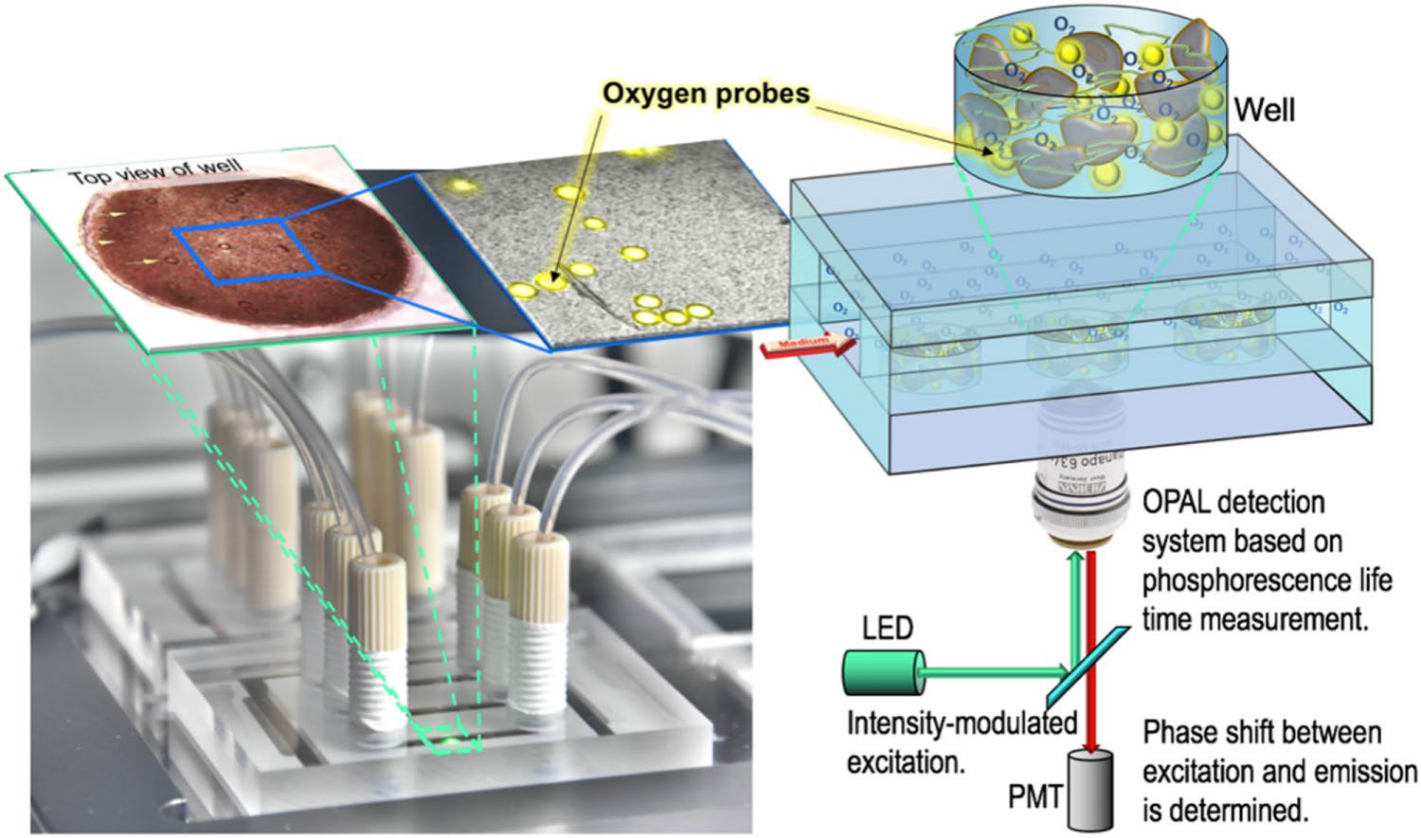
The figure shows details of the key components of the microreactor (bottom left). The reactor houses three channels (width 2 mm, height 600 µm) each equipped with three wells (diameter 1.5 mm, depth 300 µm). The wells (micrographs top left, scheme top right) accommodate the hepatocytes and the oxygen microsensor beads (in yellow) in a collagen matrix. The beads contain Ruthenium-Phenanthroline as dye. Its phosphorescence lifetime is a function of the oxygen concentration. Each of the nine wells can be individually addressed by the optical detection system using a computer controlled microscope stage. The scheme was created using PowerPoint for Mac (version 16.16.22, www.microsoft.com) and Adobe Photoshop CS6 for Mac (version 13.0 × 64, www.adobe.com).

Figure 1 shows the important components of the setup. The microbioreactor is equipped with three microfluidic channels which can be individually used. In the bottom of each channel three wells are incorporated which accommodate the cells together with the sensor beads, both embedded in a collagen matrix. Four reactors can be positioned on a motorised microscope stage such that 36 wells can be approached by the detection optics one after another. Typically, a few wells are only filled with sensor beads in the collagen matrix in order to control the oxygen concentration in the medium. For the medium supply syringe pumps are used in the suction mode with individual reservoirs for each channel. The entire setup is automated such that detection software, software for positioning the microscopy stage and for running the pumps are fully synchronised.

**Figure 2:**
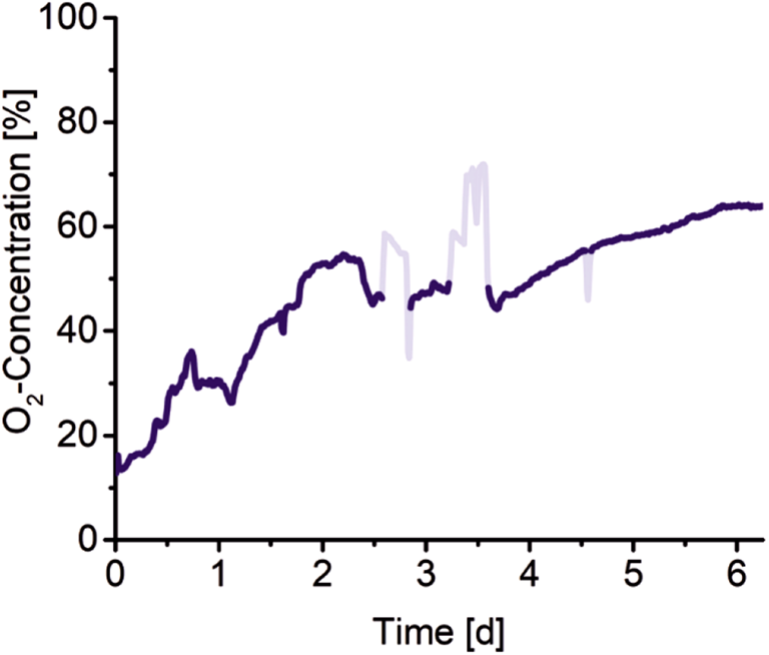
The graph shows the oxygen concentration in a well in the reactor which is filled with primary mouse hepatocytes as a function of time. The increasing oxygen concentration indicates a decreasing oxygen uptake of the cells. The peaks and dips in light blue are caused by air bubbles.

The time course of oxygen concentration in a well filled with primary cells is depicted in Fig. 2. The graph starts at an oxygen concentration of 15% relative to 100% present in the medium corresponding to oxygen saturation. This means that 85% of oxygen is consumed by the primary cells. An oxygen concentration of 15% corresponds to approximately 30 µM oxygen in the medium. This value is in the concentration range that is present in the liver (perivenous 30–50 µM, periportal 80–90 µM). The constant rise of the oxygen concentration in this well indicates decreasing oxygen consumption by the cells with time. Two different phases can be identified, a fast decrease rate lasts for 50 h and is followed by slower one afterwards. For this behaviour, three causes are conceivable: Firstly, there is a population of cells that are particularly sensitive and fragile, which easily dies and the more robust cells survive at least 6 days of recording. Secondly, the removal of the cells from the mouse, their preparation and their introduction to the reactor cause a lot of stress for the cells and may lead after some time to an adaption to the new environment, which may be visible through a reduced oxygen consumption of the cells. Thirdly, it is reported that hepatocyte dedifferentiation progresses ex vivo during prolonged cultivation^33^. The resulting altered gene expression can have a major influence on cell metabolism and thus on oxygen consumption.

**Figure 3:**
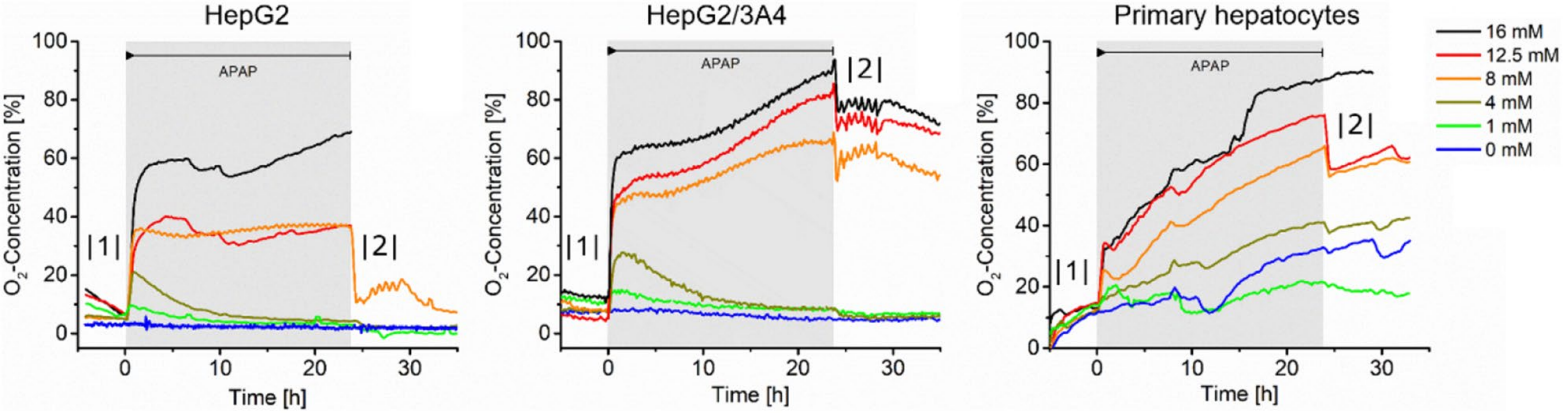
The graphs show the response of three different hepatocytes to the exposure of various concentrations of acetaminophen (period of exposure of 24 h is indicated by grey background colour). All temporal response patterns show the same characteristic features, namely a fast initial increase of the oxygen concentration in the wells (|1|), followed by much lower increase rates (in a few cases some recovery occurs) and eventually a fast drop of oxygen concentration (|2|) when acetaminophen is withdrawn from the cells. The latter indicates some recovery of the oxygen consumption of the cells.

Figure 3 provides information of the oxygen consumption of three cell types as a function of time when exposed to various concentrations of acetaminophen for 24 h (greyish region). Acetaminophen itself has no influence on the oxygen measurements (Supplementary Fig. S1). Each curve shows the recording of the oxygen concentration in a well filled with a particular cell type. The sets of graphs show a number of characteristic features common to each cell type. Most prominent is the steep increase of oxygen concentration immediately when acetaminophen is added (|1|). This is due to a step-like reduction of oxygen uptake of the cells. The step height increases with increasing drug concentration. Only in the case of primary cells being exposed to the two lower concentrations of acetaminophen hardly any steep rise is visible.

The next phase of the cellular response is dominated by less conspicuous behaviour. In the case of HepG2 and HepG2-3A4 cells at low drug concentrations (1 mM and 4 mM) after a decrease of oxygen uptake the curves again approach the baseline after a few hours. High concentrations of drug cause either no change or moderate rates of increase of oxygen levels during this period of the time course. The primary cells behave somewhat different.

Oxygen uptake decreases constantly in this part albeit not as fast as during the initial phase. When acetaminophen is withdrawn from the medium some levels of fast recovery occur in nearly all cases (|2|). Only when the primary cells are exposed to 16 mM acetaminophen no recovery is visible. In this case, all cells in the well seem to have died, in accordance what has been reported in previous work^13^.

**Figure 4:**
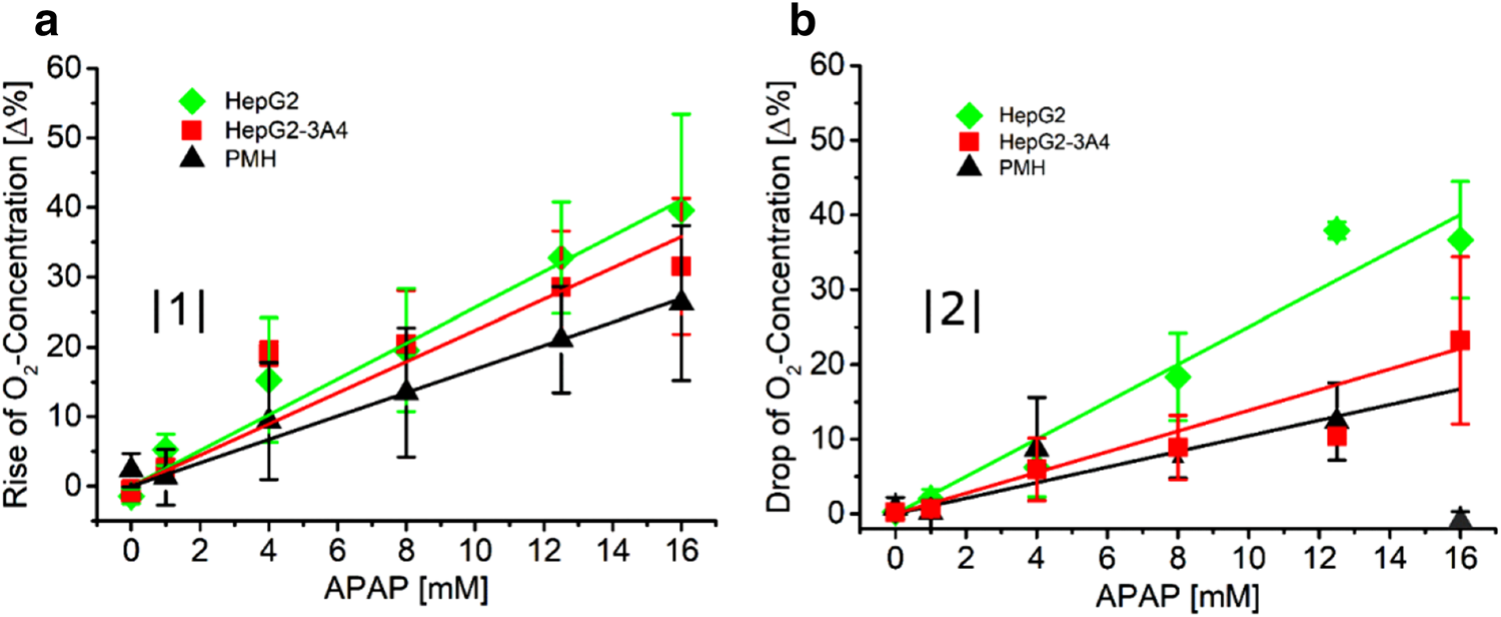
Magnitudes of fast oxygen rise |1| in the wells at the beginning of the acetaminophen exposure (**A**) and levels of recovery of oxygen concentration |2| in the wells when acetaminophen is withdrawn (**B**) as a function of acetaminophen concentration for HepG2 cells, HepG2-3A4 cells and primary mouse hepatocytes. While the increase of magnitudes of fast oxygen rise is for all cell types similar, the level of recovery of HepG2 cells is considerably higher than for the other two cell types.

In Fig. 4A the magnitudes of the fast oxygen rise are depicted as a function of acetaminophen concentration. For all three types of hepatocytes, an almost linear increase of the magnitudes of the fast response with increasing drug concentration is observed. The amplitude of cell respiration recovery after drug withdrawal as a function of acetaminophen concentration (Fig. 4B) shows a linear increase for HepG2 cells and HepG2-3A4 cells, whereas for the primary cells such a dependence cannot be observed. By comparing, the amplitudes between fast rises and respiration recovery only for the HepG2 cells comparable levels are measured. This suggests that during an exposure time of 24 h no irreversible cell damage is occurring in the HepG2 cells. A corresponding comparison of the amplitudes of fast increase of uptake and recovery after withdrawal of the drug of the other two cell types leads to a different conclusion. These two cell types experience some irreversible damage, as the respective recoveries do not equal the amplitudes of the fast rises (compare Fig. 4A,B). Live/dead staining (Supplementary Fig. S2) confirms the differences in cell damage of HepG2 and HepG2-3A4. After exposure of 8 and 16 mM acetaminophen for 24 h, more HepG2-3A4 hepatocytes died compared to HepG2 hepatocytes. A possible explanation of this observation is derived from the knowledge of the actual cause of the toxicity of acetaminophen to hepatocytes. It has been demonstrated that acetaminophen is metabolised to *N*-acetyl-p-benzoquinone imine (NAPQI) which damages or deactivates proteins and affects the mitochondrial respiration of the cell eventually leading to apoptosis and necrosis. The three key enzymes metabolizing acetaminophen are CYP1A2, CYP2E1 and CYP3A4. Although it has been demonstrated that HepG2 cells have considerable higher expression levels of these CYP enzymes when cultivated in a perfusion reactor of similar design as our one in respect to being cultivated in standard cultivation dishes, these levels are still much lower than the expression levels of HepG2-3A4 cells (for CYP3A4 only) or even primary cells. This implies that the production of NAPQI in HepG2 cells is little and the establishment of levels toxic to the cell may take considerable time. On top, NAPQI can be deactivated by glutathione for a while depending on its availability in the cell, so even in primary cells NAPQI leads to irreversible cell damage only after a sufficient period of time. Hence, the graphs of all cell types indicate a complex response pattern. The exposure of all three cell types to acetaminophen leads to a fast reduction of oxygen uptake which has been associated to a NAPQI-independent process where acetaminophen acts directly on the respiration chain^13^. This first part of the response is linearly dependent on the concentration of acetaminophen. The slowly changing part of the response curve is determined by the production of NAPQI that eventually induces cell death. Cell death reduces the overall oxygen consumption in the wells. The level of recovery of the cells after acetaminophen is withdrawn depends on the amount of acetaminophen that has been metabolised to NAPQI by the cells. We believe that this produces further evidence for the existence of two different toxic modes of action of acetaminophen, one is induced by the production of a toxic metabolite, and the other one directly affects the mitochondrial respiration.

**Figure 5:**
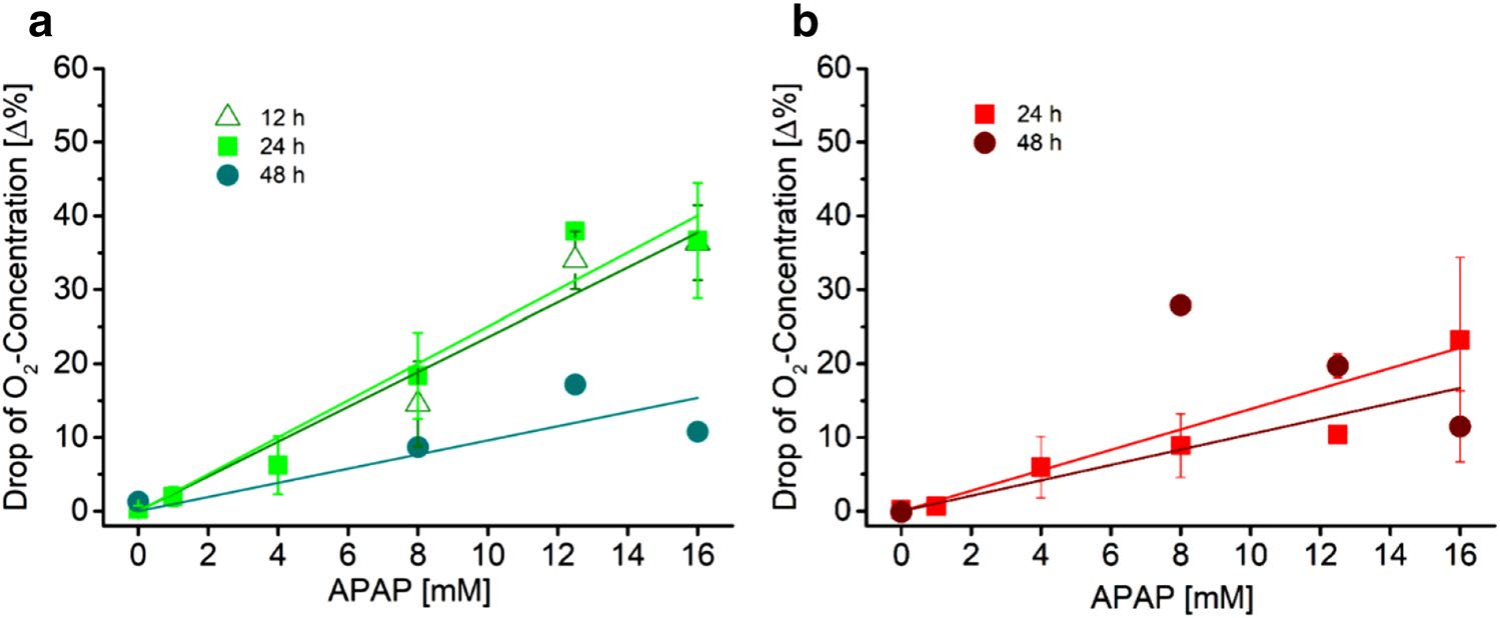
Levels of recovery of oxygen concentration |2| in the wells when acetaminophen is withdrawn as a function of acetaminophen concentration for HepG2 cells for three different times of exposure to acetaminophen (**A**). Levels of recovery of oxygen concentration |2| in the wells when acetaminophen is withdrawn as a function of acetaminophen concentration for HepG2-3A4 cells for 24 h and 48 h of exposure to acetaminophen (**B**).

This conclusion is also supported by data shown in Fig. 5. The graphs depict the level of recovery of oxygen uptake of the cells after the supply of acetaminophen has been stopped as a function of acetaminophen concentration for various periods of acetaminophen exposure. First, we have a look on the data obtained with the HepG2 cells. While for 12 and 24 h of exposure time, the graphs indicate a complete recovery of the cells after acetaminophen is withdrawn, for 48 h of exposure the level of recovery of the metabolic activity of the cells has markedly decreased. These data also demonstrate that it may be helpful to have kinetic information available for designing experiments that are based on endpoint measurements. We will discuss this issue in more detail later on.

**Figure 6:**
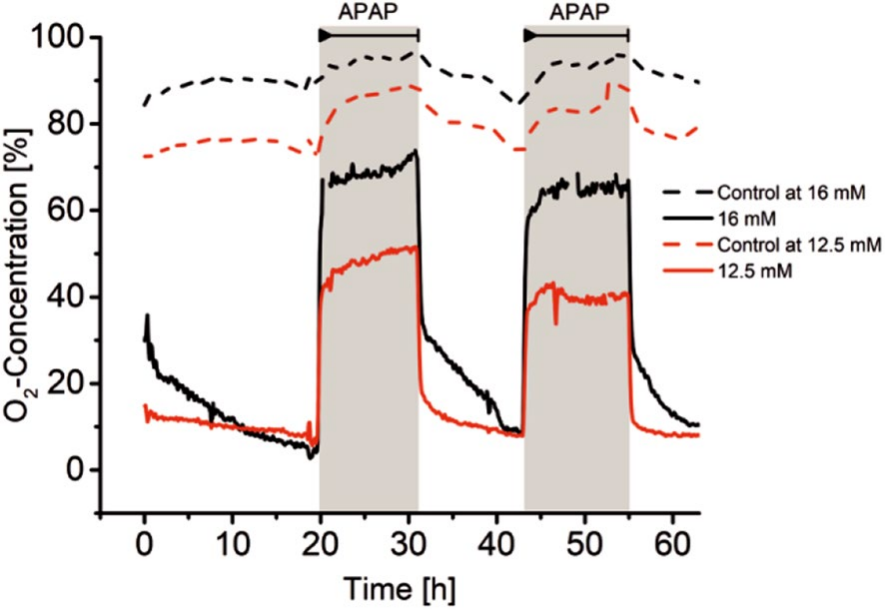
Repeated dose experiment applying two concentrations of acetaminophen (12.5 mM and 16 mM) to HepG2 hepatocytes with each exposure lasting for 12 h (period of exposure is indicated by grey background colour). After each exposure, the oxygen uptake of cells fully recovered. The data represented by the dashed line are recorded in two wells that contained only sensor beads and no cells and were located downstream (see Fig. 1) in respect to the wells containing the cells. In wells void of cell located upstream to the wells filled with cells, no decrease of oxygen concentration was observed.

Repeated dose experiments using the HepG2 cells confirm the reversibility of the fast decrease of oxygen uptake. Figure 6 depicts the response of the cells when exposed to 12.5 and 16 mM acetaminophen in a sequences of two cycles of 12 h each. An important feature of our set up is highlighted by comparing the time courses recorded when the sensor beads are positioned together with the cells in the same well (continuous line) and when the beads are separated from the cells in a different well downstream (dashed line). It is obvious that the sensitivity of the oxygen measurement dramatically drops when the sensor beads are not in the immediate vicinity of the cells. This is somewhat surprising as simulations of the flow profile show that the volume elements of the medium that pass the well containing the cells also pass to a great extend the next well downstream (data not shown). We suspect diffusive transport mainly responsible for this result. We believe that this information should be kept in mind when designing a reactor with other sensor elements that might be bulkier then the sensor beads we use, be it electrochemical sensors or optical sensors based on fibres.

**Figure 7:**
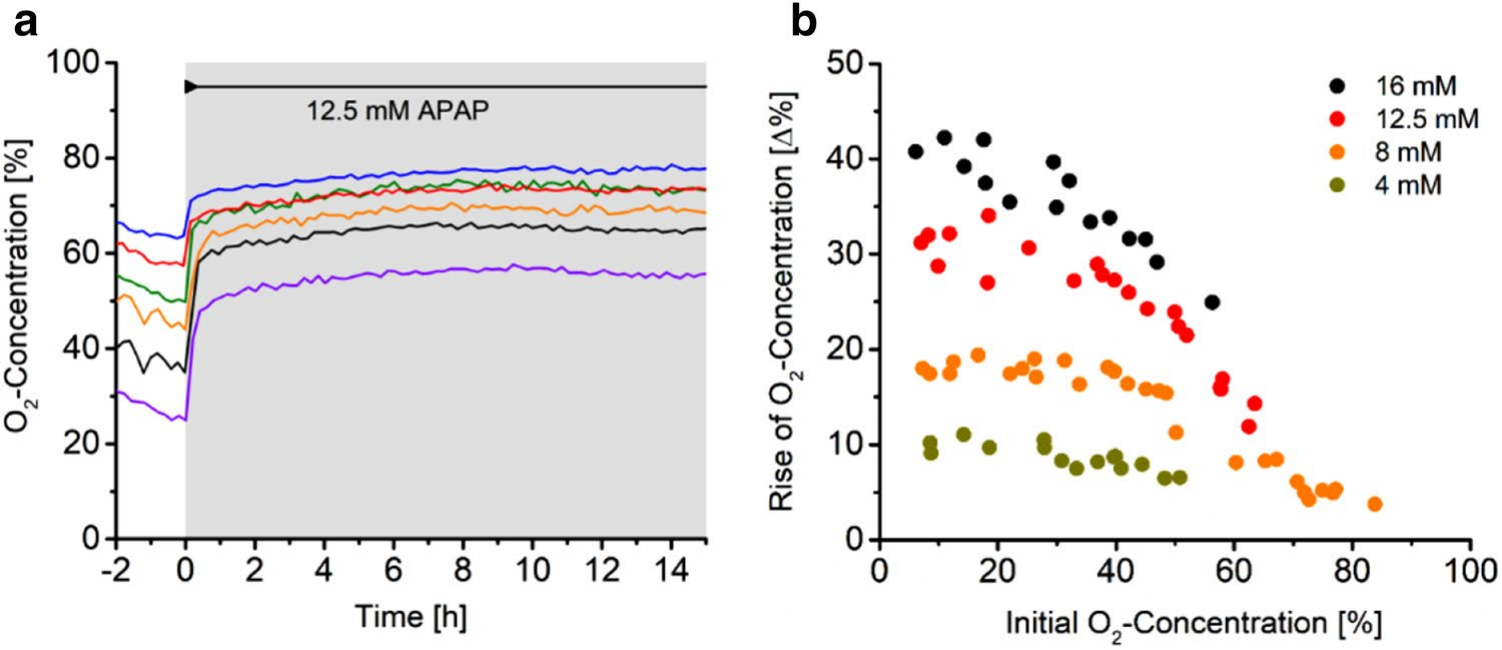
Influence of the initial oxygen level in the wells before exposure starts $\left. {{\varvec{c}}_{O2}^{cell} } \right|_{init}$ on the cellular response. (**A**) Time courses of the oxygen concentration in the wells of the reactor, which are filled with different cell numbers of HepG2 hepatocytes, during exposure to 12.5 mM acetaminophen (period of exposure is indicated by grey background colour). Varying cell numbers in the wells result in different initial oxygen concentrations. In (**B**) the magnitude of the fast cellular response (|1|) is plotted as a function of the initial oxygen level in the wells just before exposure starts $\left. {{\varvec{c}}_{O2}^{cell} } \right|_{init}$ for various concentrations of acetaminophen. The initial oxygen levels $\left. {{\varvec{c}}_{O2}^{cell} } \right|_{init}$ were adjusted by the number of cells *N* introduced to a particular well. Cell numbers *N* that correspond to an oxygen concentration close to 0% were 3 × 10^5^ cells whereas for obtaining an oxygen concentration of 80% only 5 × 10^4^ cells were introduced to one well. For initial oxygen levels $\left. {{\varvec{c}}_{O2}^{cell} } \right|_{init}$ between 0 and approximately 40% the magnitude of the fast cellular response is constant for a given acetaminophen concentration, for higher initial oxygen levels the magnitudes steadily decrease for increasing initial oxygen levels $\left. {{\varvec{c}}_{O2}^{cell} } \right|_{init}$.

The data shown in Fig. 7 address an important issue related to the measurement scheme described, namely the question, how the initial oxygen concentration in the well before the cells get in contact with the drug, affects the response of the cells to acetaminophen. Although we investigate this issue only by using acetaminophen, we believe that the results of these experiments may be very helpful when it comes to the design of tests of drugs which act on the respiration of cells. The very characteristic response curves we obtain from exposing hepatocytes to acetaminophen simplify the situation such that the response we concentrate on is fast and long-term effects related to proliferation or cell death can be easily ignored.

Figure 7A depicts time courses of the response of HepG2 cells to the exposure of 12.5 mM acetaminophen for 24 h starting at different initial oxygen levels. The magnitudes of the rise immediately after the addition of acetaminophen varies with increasing initial oxygen concentrations. The data presented in Fig. 7B are plotted as the magnitude of the initial fast rise of oxygen concentration that means of the fast drop of oxygen uptake of cells in the well when they get exposed to acetaminophen as a function of oxygen concentration in the well just before the start of the exposure $\left. {{\varvec{c}}_{{{\varvec{O}}2}}^{{{\varvec{cell}}}} } \right|_{{{\varvec{init}}}}$. This oxygen concentration is inversely related to the number of cells in the well, higher number of cells require more oxygen and vice versa. Data for various concentrations of acetaminophen are shown from 1 to 16 mM with the former corresponding to physiological values. Two different regimes of dependence are visible for all concentrations tested. From 0 to approximately 40% oxygen concentration, corresponding to cell numbers between 3 × 10^5^ and 8 × 10^4^ per well, the magnitude of the response remains constant and does not depend on the initial oxygen concentration $\left. {{\varvec{c}}_{{{\varvec{O}}2}}^{{{\varvec{cell}}}} } \right|_{{{\varvec{init}}}}$. Above 40% oxygen concentration the magnitude of the response linearly decreases with increasing oxygen concentration in the well. The value of $\left. {{\varvec{c}}_{{{\varvec{O}}2}}^{{{\varvec{cell}}}} } \right|_{{{\varvec{init}}}}$ where the transitions from the constant response to the linearly decreasing response occurs slightly shifts with the acetaminophen concentration. Although the data show some scatter, by taking advantage of the ability of our set up to collect data in a highly parallel fashion, we can produce a sufficient amount of data points within a reasonable period of time in order to gain control over the inherent uncertainty of the cellular system. As we demonstrate in the next paragraph, the data do not only help to obtain new insight in how the cells respond under a variable oxygen supply, at the same time, they also allow to better characterise the role of the environment of the cells. The latter is for example crucially important when data obtained in different laboratories to be compared. In order address this issue, we present a simple analysis of the oxygen supply to and the consumption by the cells in the wells. But before this analysis it is instructive to shortly compare the oxygen concentrations that can be established in the reactor with those present in vivo. Oxygen concentrations in the periportal and in the perivenous zones are 80–90 µM and 30–50 µM, respectively^34^. This compares to a saturated oxygen concentration (100%) in the wells of our reactor at 37 °C to approximately 200 µM (this holds for distilled water, for salt solutions, the values are a few per cent lower). Hence, oxygen concentrations between 15 and 50% correspond to physiological conditions in the liver.

We start with the assumption that the oxygen concentration at the position of the cells ${\varvec{c}}_{{{\varvec{O}}2}}^{{{\varvec{cell}}}}$ (and the sensor beads) in the well immediately before and immediately after the sharp decrease of oxygen uptake when acetaminophen is added is time independent on the relevant time scale. Then the transport of O_2_ from the medium in the channel to the cells must equal the consumption of O_2_ by the cell. Transport is driven by the concentration gradient of O_2_ between the medium ${\varvec{c}}_{{{\varvec{O}}2}}^{{{\varvec{med}}}}$ and at the location of the cells ${\varvec{c}}_{{{\varvec{O}}2}}^{{{\varvec{cell}}}}$ and can be expressed by the diffusion current

$${\varvec{I}}_{{{\varvec{O}}_{2} }} = {\varvec{k}} \times \left( {{\varvec{c}}_{{{\varvec{O}}_{2} }}^{{{\varvec{med}}}} - {\varvec{c}}_{{{\varvec{O}}_{2} }}^{{{\varvec{cell}}}} } \right)\;{\text{with}}\;{\varvec{k}} = \frac{{{\varvec{DA}}}}{{\varvec{b}}}$$

*D* is the diffusion constant of oxygen in the medium, *A* and *b* stand for the cross section and the depth of a well, respectively, although they do not necessarily describe the real dimensions of a well. They should rather be taken as geometrical parameters. The oxygen consumption by the cells in a well is simply given by

$${\varvec{V}}_{{{\varvec{O}}_{2} }} = {\varvec{N}} \times {\varvec{v}}_{{{\varvec{O}}_{2} }}$$

*N* represent the number of cells in a well and ${\varvec{v}}_{{{\varvec{O}}2}}$ describes the oxygen consumption rate in molecules per second for one cell. By equalling the expressions for ${\varvec{I}}_{{{\varvec{O}}_{2} }}$ and for ${\varvec{V}}_{{{\varvec{O}}_{2} }}$, ${\varvec{I}}_{{{\varvec{O}}_{2} }} = {\varvec{V}}_{{{\varvec{O}}_{2} }} ,\user2{ }$ one easily obtains the oxygen concentration at the position of the cells (and the sensor beads) as a function of the number of cells in a well:

$${\varvec{c}}_{{{\varvec{O}}_{2} }}^{{{\varvec{cell}}}} = {\varvec{c}}_{{{\varvec{O}}_{2} }}^{{{\varvec{med}}}} - \frac{{{\varvec{N}} \times {\varvec{v}}_{{{\varvec{O}}_{2} }} }}{{\varvec{k}}}$$

**Figure 8:**
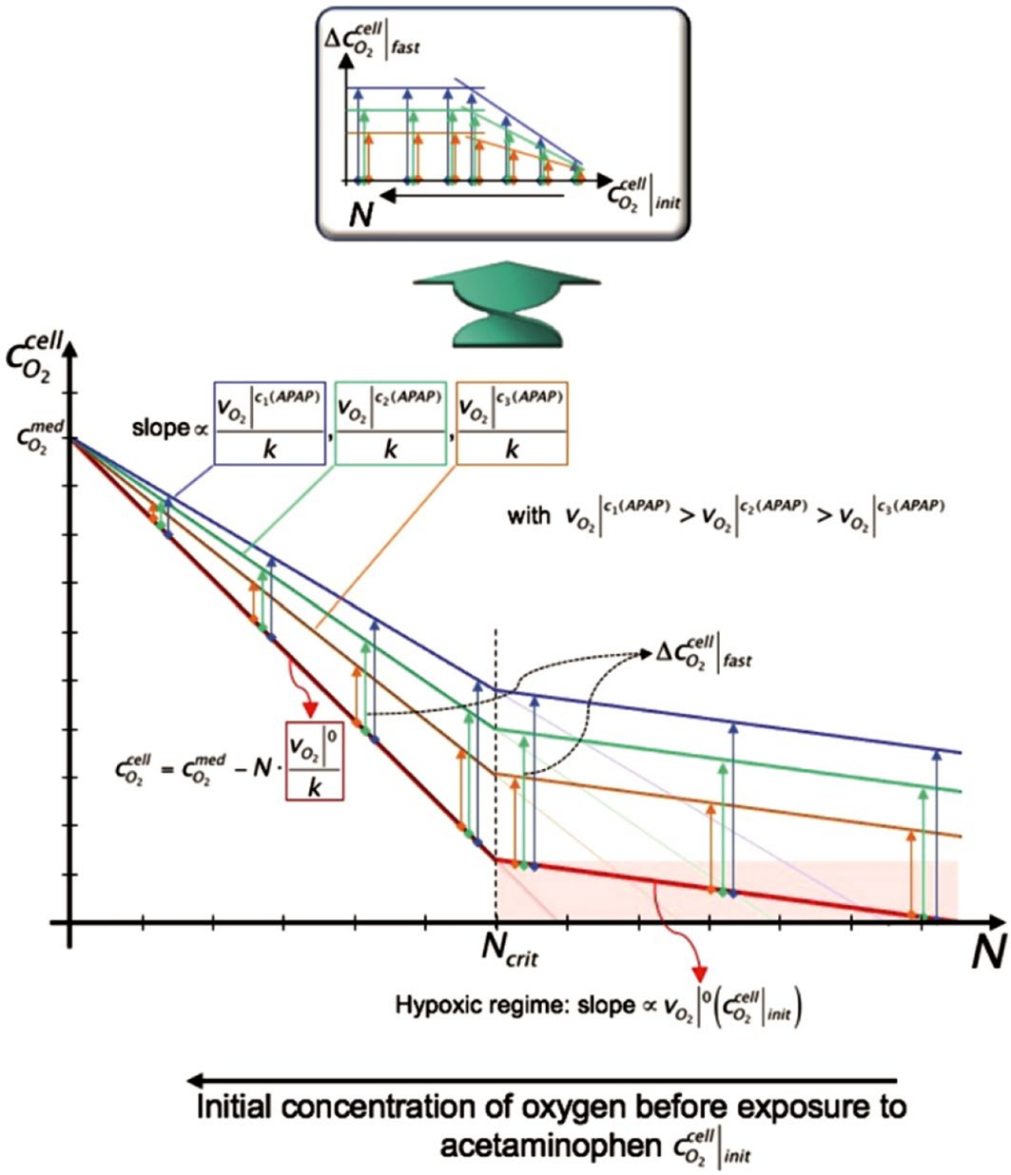
Diagram that illustrates our approach for explaining the correlation between the initial availability of oxygen to the cells $\left. {{\varvec{c}}_{O2}^{cell} } \right|_{init}$ and the magnitudes of their fast response to acetaminophen (|1|). The twisted arrow and the insert indicates that the parameter ***N*** of the abscissa of the graph is inversely related to the initial oxygen concentration of graph 7B, which shows the experimental results. The details of the diagram are described in the text. The figure was drawn using PowerPoint for Mac (version 16.16.22, www.microsoft.com).

Based on this simple model, a linear dependence of ${\varvec{c}}_{{{\varvec{O}}2}}^{{{\varvec{cell}}}}$ from *N* is derived. In a graphical representation of this equation, straight lines with the slope ${\varvec{v}}_{{{\varvec{O}}2}} /{\varvec{k}}$ cross the abscissa at ${\varvec{k}}/{\varvec{c}}_{{{\varvec{O}}2}}^{{{\varvec{med}}}} {\varvec{v}}_{{{\varvec{O}}2}}$ and the ordinate at ${\varvec{c}}_{{{\varvec{O}}2}}^{{{\varvec{med}}}}$ (see Fig. 8). Now, the oxygen consumption rate of a cell ${\varvec{v}}_{{{\varvec{O}}2}}$ varies with the concentration of acetaminophen and a bundle of straight lines can be drawn for different levels of oxygen consumption with the line with the steepest slope $\left. {{\varvec{v}}_{{{\varvec{O}}2}} } \right|^{0} /{\varvec{k}}$ representing cells that are not exposed to acetaminophen. In this picture the magnitude of the fast increase of oxygen uptake $\left. {\Delta {\varvec{c}}_{{{\varvec{O}}2}}^{{{\varvec{cell}}}} } \right|_{{{\varvec{fast}}}}$ by the cells when acetaminophen is added is represented by the vertical distance (for a given cell number *N*) between the straight line with slope $\left. {{\varvec{v}}_{{{\varvec{O}}2}} } \right|^{0} /{\varvec{k}}$ and a line described by an oxygen consumption rate $\left. {{\varvec{v}}_{{{\varvec{O}}2}} } \right|^{{{\varvec{c}}_{{\varvec{n}}} \left( {{\varvec{APAP}}} \right)}}$ of cells when exposed to acetaminophen at a concentration *c*_*n*_*(APAP)*. When comparing these graphs with the graphs obtained from the experimental data, it is clear that the former do not describe the entire set of data. The range of $\left. {{\varvec{c}}_{{{\varvec{O}}2}}^{{{\varvec{cell}}}} } \right|_{{{\varvec{init}}}}$ in which $\left. {\Delta {\varvec{c}}_{{{\varvec{O}}2}}^{{{\varvec{cell}}}} } \right|_{{{\varvec{fast}}}}$ remains constant, requires an extension such that it describes the entire set of data. For this, it is helpful to look at the situation when the number of cells in the well *N* increases and cells are not able to consume the amount of oxygen $\left. {{\varvec{v}}_{{{\varvec{O}}2}} } \right|^{0}$ they require, simply because there is not enough oxygen available. This implies that for *N* > *N*_*crit*_ hypoxic conditions prevail at the cells. In the graph this is represented as a straight line with a slope which is lower as $\left. {{\varvec{v}}_{{{\varvec{O}}2}} } \right|^{0} /{\varvec{k}}$ as $\left. {{\varvec{v}}_{{{\varvec{O}}2}} } \right|^{0} \left( {\left. {{\varvec{c}}_{{{\varvec{O}}2}}^{{{\varvec{cell}}}} } \right|_{{{\varvec{init}}}} } \right)$. Hence the slope dependents on the availability of oxygen before the cells are exposed to acetaminophen, represented by $\left. {{\varvec{c}}_{{{\varvec{O}}2}}^{{{\varvec{cell}}}} } \right|_{{{\varvec{init}}}}$. This means that the oxygen uptake of the cells $\left. {{\varvec{v}}_{{{\varvec{O}}2}} } \right|^{0} \left( {\left. {{\varvec{c}}_{{{\varvec{O}}2}}^{{{\varvec{cell}}}} } \right|_{{{\varvec{init}}}} } \right)$ steadily decreases such that $\left. {{\varvec{v}}_{{{\varvec{O}}2}} } \right|^{0} \left( {\left. {{\varvec{c}}_{{{\varvec{O}}2}}^{{{\varvec{cell}}}} } \right|_{{{\varvec{init}}}} } \right) < \left. {{\varvec{v}}_{{{\varvec{O}}2}} } \right|^{0}$. Now, if it is assumed that in this range, the response of the cells to the exposure of acetaminophen is such that the decrease in oxygen uptake by the cell simply adds up to its initial consumption, then this would show up in the graphical representation of the model as lines parallel to the one described by the slope $\left. {{\varvec{v}}_{{{\varvec{O}}2}} } \right|^{0} \left( {\left. {{\varvec{c}}_{{{\varvec{O}}2}}^{{{\varvec{cell}}}} } \right|_{{{\varvec{init}}}} } \right)$. At *N*_*crit*_, they touch the linear graphs with slopes $\left. {{\varvec{v}}_{{{\varvec{O}}2}} } \right|^{{{\varvec{c}}_{{\varvec{n}}} \left( {{\varvec{APAP}}} \right)}} /{\varvec{k}}$ describing the cellular uptake $\left. {{\varvec{v}}_{{{\varvec{O}}2}} } \right|^{{{\varvec{c}}_{{\varvec{n}}} \left( {{\varvec{APAP}}} \right)}} /{\varvec{k}}$ for a given acetaminophen concentration *c*_*n*_*(APAP)* not restricted by a limiting oxygen supply.

## Discussion

The control of all relevant conditions of in vitro tests of toxicity is a crucial issue for a couple of reasons. Firstly, the most obvious one is derived from the demands of reproducibility. Secondly, comparability of the conditions of tests performed in different labs is not always straightforward to maintain. This is a consequence of different designs of test formats, which all have some “hidden” parameters that are hard to characterise but which may be crucial for the outcome of tests. An example for such a parameter is the access of compound to be tested to the cells. Depending on the flow and transport as well as the size and density of cell sample, different parts of the sample may be exposed to different local concentrations of the compound. This may be reduced by a careful design of the fluidic environment and in addition by choosing rather small sample sizes. But the latter implies that highly sensitive probes are available such as the microsensor particles used in this work. However, sensitive sensor elements are only part of the solution for achieving high sensitivity. Close proximity of cells and sensors are vital as minute changes of parameters related to the metabolism of the cells may only be reliably measured in their microenvironment. This is demonstrated by the results shown in Fig. 6. From the time courses obtained from a repeated dose experiment it is clearly visible that the sensor elements positioned just a few millimetres downstream produce a much lower signal from a given change of oxygen uptake of the cells than sensors in close proximity to the cells. In this respect, it is also worth mentioning that the reduction of the size of the cell sample has its inherent limits simply because of the finite sensitivity of the sensor beads^26^. At present, this excludes the use of these oxygen microsensor beads for highly miniaturised chip formats. But on the other hand, in our view, there are hardly any clear advantages of reducing the dimensions of the chips to the technological limits. We believe that depending on the questions to be addressed an optimised size regime for the design of a device in respect to a proper performance should be the goal rather than minimising the system to its limits. This implies for our scenario that the supply of cells, the cellular uptake of oxygen and the sensitivity of the sensor elements must be in a particular balance in order to obtain meaningful results. The fact that the readout of the microsensors is based on the detection of visible light provides considerable freedom for the design of a reactor in respect to the geometry of the fluidic components and of the materials as long as they are sufficiently transparent. In addition, as the detection scheme is based on the measurement of phosphorescence lifetime, no focussing of the sensor particles is required, and their positioning tolerates some degree of randomness.

The most popular formats for in vitro tests for the assessment of the toxicity of compounds are based on endpoint measurements, although they only provide a rather limited amount of information. This is the consequence of the impact of a number of factors, namely the lack of alternatives, the relative ease and straightforwardness of setting up these formats, mechanistic models that would require substantially more information to be tested are not available and hence the content of information provided may be sufficient^35^. Finally, many endpoint formats are very well established in the field as they are validated and correlated to in vivo tests. In most scenarios, they form an important part of a broader assessment of toxicity^36^ and the motivation to replace them by more informative methods is rather little. Unfortunately, extending endpoint experiments to formats that allow kinetic data generation is in most cases time-consuming and labor-intensive, since an invasive analysis setup is integrated. This scenario leads to the somewhat unfortunate situation that new innovative formats, which provide a much richer set of information, get stuck in the process of developing novel powerful in vitro tests. In order to overcome this deadlock, we propose to make use of the potential of kinetic measurements for the determination of the time points that should be used in endpoint assays for gathering the most significant data.

It has already been demonstrated that the continuous monitoring of the oxygen uptake of hepatocytes in real time provides valuable data on the kinetics of their response that helped to identify and to characterise a previously unknown mode of action of the hepatotoxic compound acetaminophen^13^. In order to derive the most meaningful information from the measurement, it is therefore necessary to determine suitable time points within the various time courses. In the first place, the possibility of the method described to obtain information on the cellular response from a locally well defined sample in real time minimised the risk of generating artefacts when cell samples are taken in order to identify or measure cell markers using invasive methods. In particular, it allows a precise timing of sample collection and hence helps to establish a strong correlation between kinetic data and possible time points. Secondly, the evaluation of a set of time courses of the cellular response helps to identify the time point or set of time points that contain the most valuable and significant information of the process. In this context, the monitoring of the oxygen consumption of hepatocytes in response to being exposed to acetaminophen in real time provides a very informative example for making the case for this approach.

An instructive example of this reasoning can be derived from the data shown in Figs. 3 and 5. The graphs shown in Fig. 3 include two different kinetics suggesting two modes of action of acetaminophen. The fast response would be hardly visible using end-point measurements as it is unlikely that using this approach a sufficient temporal resolution can be obtained. Next, it is obvious that it is very important to control whether the cells are still exposed to acetaminophen or how long the delay is between removal of acetaminophen and the start of, for example, a staining procedure of the cells for the identification of a certain marker. If kinetic data suggest that more than one mode of action is induced by the compound, this information may be important for the selection of a particular cell marker for the characterisation of the process of interest. The specificity of cell markers to a particular process can be evaluated or maintained by trying to choose end-points with variable contributions of the different mode of actions. The data in Fig. 5 could be used to identify endpoints for the determination of TC50 (toxic concentration) or LC50 (lethal concentration) values. Although it is obvious that the duration of exposure is a crucial parameter for the determination of these values, it is often not easy to derive most relevant time points if the response pattern is complex. Here kinetic data may help to make an appropriate choice. This particularly applies when it comes to comparative studies using different cell models.

Finally, we like to emphasise the utility of continuously monitoring cellular kinetics for the characterization of cellular dynamics. Although, the system chosen has no direct clinical relevance as far as the toxicity of acetaminophen is concerned^37–39^, its two response mechanism in different time regimes allowed us to highlight the virtues of the new technology. We believe that by complementing end point measurements with methods that provide information on the dynamic behaviour of cells, two goals can be achieved: Firstly, the quality of endpoint measurements can be improved by, for example, a precise identification of suitable time points. Secondly, a validation of in vitro test systems that produce kinetic data can be obtained “en passant”. This may eventually lead to an acceleration of the acceptance of these systems such that the wealth of information can be fully exploited for the advancement of the field of toxicology.

## Conclusions

In this contribution, we describe an in vitro test system for the continuous monitoring of the oxygen uptake of cells in real time when they are exposed to toxic compounds. In a microreactor up to nine cell samples are accommodated and continuously perfused by the medium and any substance of interest. Microsensor beads allow the measurement of the oxygen concentration in the immediate vicinity of the cells. By adjusting medium flow and sample size, this information can be exploited for the determination of the cellular oxygen uptake, which can serve as a measure for the metabolic activity of the cells. The response of three types of hepatocytes is thoroughly analysed when acetaminophen at various concentrations is used as a test compound. The cells differ by their capacity to metabolise acetaminophen. This has been used to produce further evidence for the existence of two different toxic modes of action of acetaminophen, one is induced by the production of the known toxic metabolite NAPQI, and the other one directly affects the mitochondrial respiration and has only recently been identified using this technology. By making use of the characteristic response pattern of the cells induced by acetaminophen, a detailed analysis of the influence of the mode of application of the compound and of the role of the microenvironment of the cells on the cellular response has been performed. These results may help to refine endpoint measurements, which are still standard in the field of toxicology. Moreover, via this route the establishment of this novel method for the assessment of the toxicity of compounds in the field appears to be conceivable.

In addition, studies of the processes of vascularization in tumors, as described by Nashimoto et al.^40^, which are induced by hypoxia, can be monitored more precisely through the possibility of determining the oxygen concentration directly in the vicinity of the cells, as shown here.

## Supplementary information

Supplementary Information
