# Supplementary material for: Real time monitoring of oxygen uptake of hepatocytes in a microreactor using optical microsensors
Source: Sci Rep. 2020 Aug 13;10:13700. doi: 10.1038/s41598-020-70785-6 (PMC7426412; doi:10.1038/s41598-020-70785-6)
Supplement: Supplementary file 1 — Supplementary Information [file 41598_2020_70785_MOESM1_ESM.pdf]

Supplementary Information

Real time monitoring of oxygen uptake of hepatocytes in a microreactor using optical microsensors

Christian Gehre [1], Marie Flechner [1], Sarah Kammerer [2], Jan-Heiner Küpper [2], Charles Dominic Coleman [3], Gerhard Paul Püschel [3], Katja Uhlig *[1] and Claus Duschl [1]

1 Branch Bioanalytics and Bioprocesses (IZI-BB), Fraunhofer-Institute for Cell Therapy and Immunology, Potsdam, Germany. *Correspondence to Katja.Uhlig@izi-bb.fraunhofer.de

2 Faculty of Science, Brandenburg University of Technology Cottbus-Senftenberg, Senftenberg, Germany.

3 Department of Nutritional Biochemistry, Institute of Nutritional Science, University of Potsdam, Nuthetal, Germany.

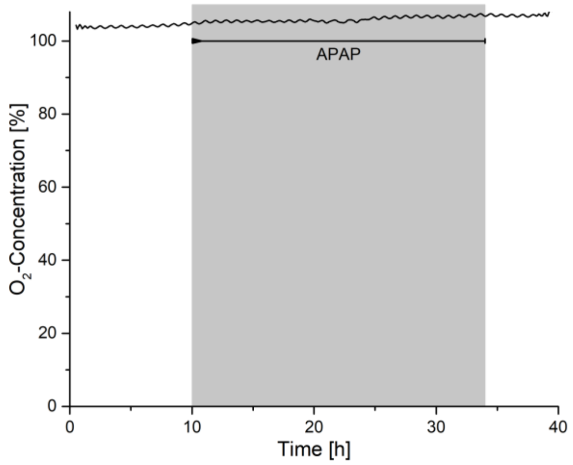

Supplementary Figure S1: Influence of acetaminophen on oxygen measurements. To exclude interference of the test substance, oxygen sensor particles were embedded in collagen in the cavities of a microreactor channel. Subsequently, medium without acetaminophen was pumped through the channel for 10 h, followed by medium with 16 mM acetaminophen for 24 h, followed by medium without acetaminophen for 6 h. The oxygen concentration was measured simultaneously. The oxygen measurements show that acetaminophen does not affect the oxygen sensor system.

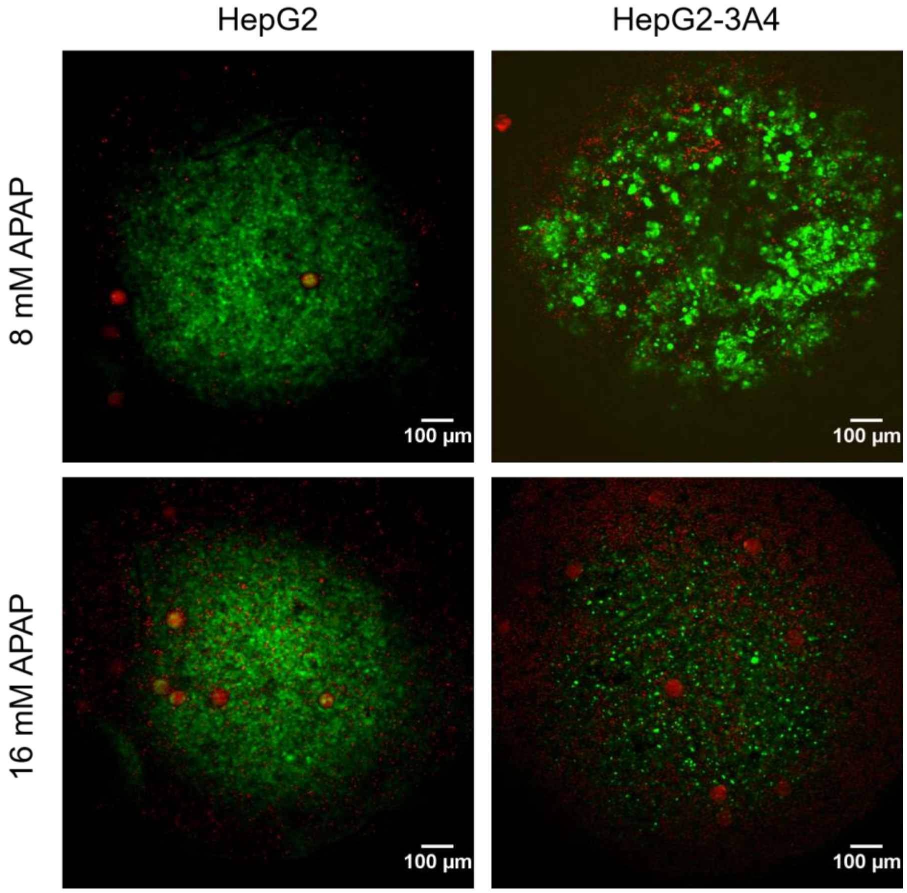

Supplementary Figure S2: Confocal microscopy images of HepG2 and HepG2-3A4. After 24 h exposure of 8 and 16 mM acetaminophen in the microreactor, living (green) and dead (red) cells were stained to investigate the toxicity of acetaminophen. The oxygen sensors are also displayed in red.

Cell staining and image acquisition: The hepatocytes were washed with PBS and then incubated with 6 µM Calcein AM (Life Technologies, USA) and 5 µM Propidium iodide (Sigma-Aldrich, USA) in cell culture medium for 30 minutes at 37 °C and 5 % CO_2 until microscopic examination. For visualisation a confocal laser scanning microscope (510 Meta, Zeiss, Oberkochen, Germany) equipped with an argon laser and a 10 x / 0.3 objective was employed. For image acquisition, the pinhole was set to 1 Airy unit (image slice of approximately 13 µm) and the samples were scanned in different focal planes using an interval of 5.7 µm. The image were processed with ImageJ using maximum intensity projection.
